# Insights into epidemiological trends of severe chest injuries: an analysis of age, period, and cohort from 1990 to 2019 using the Global Burden of Disease study 2019

**DOI:** 10.1186/s13049-024-01258-2

**Published:** 2024-09-16

**Authors:** Qingsong Chen, Guangbin Huang, Tao Li, Qi Zhang, Ping He, Jun Yang, Yongming Li, Dingyuan Du

**Affiliations:** 1 School of Microelectronics and Communication Engineering of Chongqing University, Chongqing University Central Hospital (Chongqing Emergency Medical Center), No. 174, Zhengjie street, Shapingba District, 400044, and No. 1, Jiangkang Road, YuzhongDistrict, 400014, Chongqing, China; 2 Department of Traumatology, National Regional Trauma Medical Center, Chongqing University Central Hospital (Chongqing Emergency Medical Center), No. 1, Jiangkang Road, Yuzhong District, 400014 Chongqing, China

**Keywords:** Severe chest injury, Incidence, Prevalence, Years lived with disability, Causes, Age-period-cohort, ARIMA model, Global Burden of Disease 2019

## Abstract

**Background:**

This study assessed the global trends and burden of severe chest injury, including rib fractures, lung contusions, and heart injuries from 1990 to 2019. Herein, we predicted the burden patterns and temporal trends of severe chest injuries to provide epidemiological evidence globally and in China.

**Methods:**

In our analysis, the age-standardized incidence rate (ASIR), prevalence rate (ASPR), and years lived with disability rate (ASYR) of severe chest injury were analyzed by gender, age, sociodemographic index, and geographical region between 1990 and 2019 using data from the Global Burden of Disease study 2019. Trends were depicted by calculating the estimated annual percentage changes (EAPCs). The impact of age, period, and cohort factors was assessed using an Age-Period-Cohort model. Autoregressive integrated moving average (ARIMA) model was employed to predict severe chest injury trends from 2020 to 2050.

**Results:**

In 2019, the global number of severe chest injury cases reached 7.95 million, with the highest incidence rate observed in Central Europe (209.61). Afghanistan had the highest ASIRs at 277.52, while North Korea had the lowest ASIRs at 41.02. From 1990 to 2019, the Syrian Arab Republic saw significant increases in ASIR, ASPR, and ASYR, with EAPCs of 10.4%, 9.31%, and 10.3%, respectively. Burundi experienced a decrease in ASIR with an EAPC of − 6.85% (95% confidence interval [CI] − 11.11, − 2.37), while Liberia’s ASPR and ASYR declined with EAPCs of − 3.22% (95% CI − 4.73, − 1.69) and − 5.67% (95% CI − 8.00, − 3.28), respectively. Falls and road injuries remained the most common causes. The relative risk of severe chest injury by age, period, and cohort demonstrated a complex effect globally and in China. The ARIMA model forecasted a steady increase in global numbers from 2020 to 2050, while in China, it forecasted an increase in incidence, a decrease in ASIR and ASYR, and an increase in ASPR.

**Conclusions:**

This study provides a groundbreaking analysis of global severe chest injury, shedding light on its measures and impact. These findings highlight the need for timely, specialized care and addressing regional disparities to mitigate the severe chest injury burden.

**Supplementary Information:**

The online version contains supplementary material available at 10.1186/s13049-024-01258-2.

## Background

In recent years, significant progress has been made in the management of severe chest injuries. This progress is supported by a variety of studies, ranging from the development of damage control surgery techniques to research on pulmonary contusion in mechanically ventilated individuals following severe trauma [1, 2]. Additionally, evaluation and treatment strategies for severe chest injury have been explored [3]. These studies offer valuable insights, particularly in adapting early management strategies based on severity variations and weighing the benefits and drawbacks of utilizing extracorporeal membrane oxygenation (ECMO) support in specific patient groups [4, 5]. Operative stabilization of chest wall trauma, including the use of rib plating, has demonstrated substantial benefits both immediately after the injury and in the long term, helping patients remain symptom-free and achieve optimal recovery [6, 7]. The advancements in techniques and understanding of surgical indications have further improved patient recovery outcomes. Additionally, the role of emergency resuscitation thoracotomy in managing severe chest trauma has been extensively studied, highlighting its life-saving potential despite its limited applicability in certain situations [8]. Biomarkers have also become increasingly crucial in diagnosing and managing acute traumatic lung injury, enabling a more precise assessment of patient conditions and aiding in treatment planning [9].

The increasing prevalence of noncommunicable diseases poses a significant global health challenge. Global Burden of Disease (GBD) studies recommend that health systems worldwide adapt by prioritizing preventive measures and implementing robust healthcare interventions to mitigate the impact of noncommunicable diseases [10]. The GBD 2019 study report offers detailed insights into health outcomes, risk factors, and the effectiveness of health systems across 204 countries. This edition is crucial for identifying health priorities, directing resource allocation to address disparities, and improving global health outcomes [11]. Studies investigating the relationship between global health aid and disease burden reveal discrepancies in funding distribution. This highlights the necessity for a more equitable allocation of resources, informed by GBD data, to ensure that aid reaches regions with the most pressing health needs [12]. Studies on global disease epidemiology, such as ischemic heart disease and myocarditis, use GBD data to analyze prevalence, trends, and regional differences. These studies highlight the significance of targeted prevention, improved healthcare accessibility, and quality care to reduce disease burden and enhance health outcomes [13, 14].

This study aimed to collect comprehensive data on the burden of severe chest injury from the GBD study 2019. Our objective was to present age-standardized rates (ASRs), analyze the estimated annual percentage changes (EAPCs), forecast trends, and assess epidemiological patterns across various regions, countries, time frames, and demographics. To facilitate in-depth analysis, an Age-Period-Cohort Intrinsic Estimator (APC-IE) model was employed. Furthermore, we investigated the causes of severe chest injury and explored the discrepancies between China and the global landscape in terms of severe chest injury epidemiology.

## Methods

### Data sources and case definition

The GBD 2019 study assessed health loss associated with 369 diseases, injuries, and impairments, as well as 87 risk factors across 204 countries. It provided valuable insights into the global health landscape, highlighting the impact of various health conditions and risk factors. Severe chest injury classification utilized ICD-9 and ICD-10 codes, ensuring comprehensive epidemiological analysis from 1990 to 2019. Severe chest injuries include the following types: rib fractures (S22.3 and S22.4), pulmonary contusion (S27.31), hemothorax (S27.1), pneumothorax (S27.0), sternal fractures (S22.2), mediastinal injuries (S27.8), great vessel injuries (S25.0, S25.1, and S25.2), and open chest injuries (S21.2). Key data, including crude incidence, prevalence, years lived with disability (YLD), and ASRs, was sourced from the Global Health Data Exchange tool, available at https://vizhub.healthdata.org/gbd-results/. Data processing in the GBD study involved stringent standardized procedures to address biases and ensure consistency in incidence, prevalence, and YLD assessments [15, 16]. These procedures included redistributing causes of death and rectifying measurements, enhancing the study’s reliability and accuracy. The research framework analyzed severe chest injury data globally, regionally, and specifically in China, using linear regression models to explore trends from 1990 to 2019, including demographic subgroups categorized by age and gender (Additional file 2: Fig. S1).

### Estimation of incidence, prevalence, and YLD

The methods used to estimate the burden of injury in patients for the GBD 2019 study are detailed in a previously published article [15]. This study offers a comprehensive overview of the methodologies, including data sources, analytical processes, and calculations employed to estimate the severe chest injury burden. It serves as a valuable resource for understanding the specific methods employed in GBD 2019 to assess the burden of injury. The study reports on the incidence, prevalence, and YLD of severe chest injury. Incidence measures the annual rate of new severe chest injury cases per at-risk population, whereas prevalence quantifies the total number of existing and new severe chest injury cases within a year relative to the at-risk population [17]. YLD reflects the overall impact of severe chest injury on quality of life and severity. The calculations combine prevalence data with disability weights from the GBD 2019 study. The formula for calculating YLD is as follows: YLD = Prevalence × Disability Weight, where “Prevalence” represents the total number of cases and “Disability Weight” quantifies the severity of the disability on a scale from 0 to 1 [18]. Additionally, the sociodemographic index (SDI) assesses the population’s socioeconomic status by integrating income per capita, educational attainment, and fertility rates in women under 25 years of age. It is based on the United Nations’ Human Development Index (HDI) methodology, adapted for health research to compare disease burdens across different socioeconomic contexts. SDI plays a crucial role in GBD studies by analyzing how social and economic factors impact health outcomes [19].

### Age-period-cohort analysis

The Age-Period-Cohort model, using the Poisson distribution, is designed to elucidate the variations in trends of severe chest injury incidence, prevalence, and YLD by considering age, period, and cohort effects [20]. Positive effects observed for age, period, and cohort indicate potential risk factors, while negative values suggest protective factors. The intrinsic estimator (IE) method was employed to address the linear relationships among age, period, and cohort [21, 22]. The IE method is a reliable approach for resolving such issues due to its versatility and independence from conditional assumptions. Model fit assessment was conducted using the Akaike Information Criterion and the Bayesian Information Criterion [23]. The statistical analysis was conducted using STATA software (Version 16.0; StataCorp).

### ARIMA model

The autoregressive integrated moving average (ARIMA) model, which combines autoregressive and moving average components with differencing to achieve stationarity, serves as a robust method for forecasting time series data. In the ARIMA (p, d, q) notation, “p” represents the number of autoregressive terms, “d” signifies the degree of differencing required to stabilize the series, and “q” denotes the number of moving average terms. This study utilizes the ARIMA model to analyze trends in the disease burden, including incidence, prevalence, YLD, age-standardized incidence rate (ASIR), age-standardized prevalence rate (ASPR), and age-standardized YLD rate (ASYR), projecting the future burden of severe chest injury globally and specifically in China from 2020 to 2050.

### Statistical analysis

In this study, the incidence, prevalence, and YLD numbers and rates for severe chest injury were presented across various age groups and genders, along with their 95% uncertainty intervals (UIs). ASRs were used to adjust for demographic variations. Moreover, the EAPC was employed to illustrate the evolving trends of severe chest injury over time, quantifying the annual percentage change through a regression line to the natural logarithm of the rates. A positive *β* value indicated an increase in rates, while a negative *β* value indicated a decrease over time [24]. For each EAPC value, 95% confidence intervals (CIs) were presented instead of 95% UIs. An increasing ASR trend was denoted by an EAPC value below the 95% CI limit exceeding zero, while a decreasing trend was represented by an EAPC value above the 95% CI limit below zero. EAPC values with 95% CIs encompassing zero denoted a stable trend. Data visualization and statistical analyses were conducted using R software (Version 4.3.3). Pearson correlation analysis was conducted to assess the relationship between the HDI, ASIR, ASDR, and EAPC. A significance threshold of a *p*-value less than 0.05 was applied to identify statistically significant differences.

## Results

### Global burden and trends of severe chest injury

In 2019, the estimated global incidence of severe chest injury was 7,953,386 cases (95% UI 6,132,771–10,165,826) across both genders, with an ASIR of 102 per 100,000 individuals (95% UI 78.66–130.57). A notable decreasing trend in ASIR for severe chest injury was observed from 1990 to 2019, with an EAPC of − 0.41 (95% UI − 0.54, − 0.28). The prevalence estimates for 2019 revealed 1,976,282 cases (95% UI 1,659,145–2,372,715) with an ASPR of 24.88 per 100,000 individuals (95% UI 20.79–29.88). The ASPR of severe chest injury has shown a decreasing trend since 1990, as indicated by an EAPC of − 0.26 (95% CI − 0.35, − 0.17). Additionally, the YLD for severe chest injury in 2019 were estimated at 392,996 cases (95% UI 244,235–599,630), with an ASYR of 5.02 per 100,000 individuals (95% UI 3.11–7.69). The ASYR has also demonstrated a declining trend since 1990, with an EAPC of − 0.37 (95% CI − 0.49, − 0.25) (Table 1). The number of incident cases increased from 6.4 million (95% UI 4.9–8.4 million) in 1990 to 8.0 million (95% UI 6.1–10.2 million) in 2019, reflecting a 24.14% increase. Similarly, the number of prevalent cases rose from 1.3 million (95% UI 1.0–1.6 million) in 1990 to 2.0 million (95% UI 1.7–2.4 million) in 2019, reflecting a 53.14% increase. Additionally, the number of YLD cases increased from 302,179 (95% UI 181,236–458,699) in 1990 to 392,996 (95% UI 244,235–599,630) in 2019, reflecting a 30.06% increase (Table S1).

**Table 1:** The ASIR, ASPR, and ASYR with EAPC in severe chest injury from 1990 to 2019

Region	incidence (95% UI)		EAPC (95% CI)	prevalence (95% UI)		EAPC (95% CI)	YLDs (95% UI)		EAPC (95% CI)
	1990	2019		1990	2019		1990	2019	
	ASR per 100,000	ASR per 100,000		ASR per 100,000	ASR per 100,000		ASR per 100,000	ASR per 100,000	
Global	118.66 (90.89–154.47)	102 (78.66–130.57)	− 0.41 (-0.54–0.28)	26.79 (22.08–32.61)	24.88 (20.79–29.88)	− 0.26 (-0.35–0.17)	5.73 (3.48–8.67)	5.02 (3.11–7.69)	− 0.37 (− 0.49–0.25)
Africa	137.63 (84.52–240.29)	76.24 (58.21–98.45)	− 1.42 (− 1.96–0.87)	28.11 (20.6–40.53)	21.19 (17.33–26.44)	− 0.64 (− 0.91–0.36)	6.53 (3.57–11.85)	3.87 (2.43–5.74)	− 1.25 (− 1.73–0.76)
African Region	147.81 (88.87–263.02)	76.66 (58.11–99.31)	− 1.65 (− 2.29–1.02)	30.62 (22.34–44.56)	22.46 (18.41–27.99)	− 0.73 (− 1.04–0.42)	7.04 (3.79–12.97)	3.95 (2.49–5.87)	− 1.44 (− 2–0.88)
America	155.82 (116.92–204.74)	133.45 (101.2–175.33)	− 0.45 (− 0.57–0.32)	35.6 (29.5–44.24)	31.54 (26.33–38.7)	− 0.36 (− 0.46–0.25)	7.54 (4.53–11.75)	6.51 (3.94–10.2)	-0.43 (-0.55–0.31)
Andean Latin America	107.92 (77.74–158.55)	76.01 (58.11–100.86)	− 0.82 (− 1.12–0.51)	19.62 (14.57–27.64)	15.1 (11.97–19.47)	− 0.7 (− 0.87–0.52)	4.98 (2.88–8.3)	3.56 (2.14–5.56)	− 0.8 (− 1.08–0.52)
Asia	93.29 (72.58–118.27)	96.79 (74.93–123.1)	0.24 (0.09–0.4)	22.19 (18.42–26.85)	24.04 (20.06–28.87)	0.25 (0.14–0.36)	4.56 (2.83–6.85)	4.78 (3–7.22)	0.26 (0.11–0.4)
Australasia	259.68 (184.44–383.09)	247.36 (176.45–358.3)	− 0.23 (− 0.28–0.18)	45.44 (33.42–63.59)	43.94 (32.64–61.17)	− 0.17 (− 0.22–0.12)	11.86 (6.58–19.68)	11.33 (6.32–18.8)	− 0.22 (− 0.27–0.17)
Caribbean	92.35 (72.27–121.55)	99.14 (76.58–132.52)	0.62 (− 0.51–1.76)	19.11 (15.53–24.02)	22.73 (17.82–29.45)	1.03 (0.22–1.86)	4.37 (2.63–6.84)	4.79 (2.9–7.44)	0.71 (-0.38–1.82)
Central Asia	128.83 (98.66–171.46)	111.58 (83.94–151.64)	− 1.07 (− 1.54–0.59)	24.83 (19.46–32.09)	22.06 (17.32–28.71)	− 0.79 (− 1.11–0.46)	6 (3.59–9.46)	5.22 (3.09–8.3)	− 1.02 (− 1.47–0.57)
Central Europe	245.68 (181.14–343.07)	209.61 (152.09–297.79)	− 0.83 (− 1–0.66)	47.26 (36.39–62.94)	40.02 (30.73–53.43)	− 0.77 (− 0.89–0.66)	11.44 (6.62–18.57)	9.74 (5.62–16.03)	− 0.83 (− 0.99–0.66)
Central Latin America	151.56 (115.21–198.14)	121.69 (92.42–161.18)	− 0.02 (− 0.28–0.24)	33.46 (27.17–41.59)	26.53 (21.61–33.21)	− 0.01 (− 0.26–0.24)	7.27 (4.39–11.32)	5.82 (3.51–9.16)	− 0.02 (− 0.27–0.24)
Central Sub-Saharan Africa	73.6 (51.32–114.33)	53.99 (41.48–71.82)	− 2.46 (− 4.28–0.61)	16.42 (12.24–23.35)	16.01 (11.51–25.08)	− 1 (-2.12–0.13)	3.55 (2.09–6.01)	2.77 (1.74–4.17)	− 2.16 (− 3.84–0.46)
Commonwealth High Income	142.31 (106.01–195.83)	136.49 (100.89–187.77)	− 0.21 (− 0.28–0.14)	26.87 (20.8–35.28)	26.32 (20.4–34.56)	− 0.13 (− 0.18–0.07)	6.6 (3.86–10.55)	6.36 (3.74–10.27)	− 0.19 (− 0.26–0.13)
Commonwealth Low Income	81.66 (60.92–110.22)	73.28 (56.56–93.24)	− 1.31 (− 2.09–0.53)	21.02 (17.14–26.41)	19.93 (16.57–24.61)	− 0.92 (− 1.33–0.51)	4.07 (2.5–6.13)	3.7 (2.33–5.56)	− 1.24 (− 1.95–0.53)
Commonwealth Middle Income	101.44 (78.7–130.05)	95.87 (73.92–122.43)	− 0.03 (− 0.15–0.09)	23.81 (19.92–28.42)	24.56 (20.72–29.44)	0.21 (0.09–0.34)	4.94 (3.06–7.26)	4.77 (2.96–7.2)	0.03 (− 0.09–0.15)
East Asia	72.86 (53.73–99.57)	86.42 (63.71–115.38)	0.09 (− 0.26–0.45)	18.32 (15.34–22.32)	23.16 (19.7–27.78)	0.41 (0.09–0.73)	3.61 (2.22–5.53)	4.37 (2.66–6.73)	0.19 (− 0.15–0.53)
East Asia & Pacific—WB	84.9 (64.73–111.33)	89.37 (67.09–116.57)	− 0.13 (− 0.37–0.11)	20.36 (17.04–24.6)	22.43 (18.93–26.98)	0.05 (− 0.17–0.27)	4.16 (2.54–6.37)	4.44 (2.7–6.78)	− 0.07 (− 0.31–0.16)
Eastern Europe	246.75 (189.71–327.46)	189.92 (145.99–250.84)	− 0.97 (− 1.12–0.81)	49.28 (39.18–63.03)	38.58 (30.87–49.16)	− 0.88 (− 1.03–0.73)	11.58 (6.83–18.14)	8.95 (5.38–13.98)	− 0.95 (− 1.1–0.79)
Eastern Mediterranean Region	107.69 (81.71–146.33)	106.61 (77.77–154.02)	1.61 (1.03–2.18)	26.58 (18.7–42.41)	25.35 (18.22–37.8)	0.65 (0.31–1)	5.29 (3.32–8.18)	5.19 (3.12–8.27)	1.42 (0.89–1.95)
Eastern Sub-Saharan Africa	257.97 (125.49–546.66)	78.33 (59.97–100.85)	− 2.54 (− 3.59–1.48)	45.73 (27.81–78.79)	24.71 (19.08–35.67)	− 1.28 (− 1.83–0.74)	11.94 (5.59–25.82)	4.11 (2.62–6.07)	− 2.26 (− 3.2–1.31)
Europe	173.11 (130.28–231.42)	138.44 (104.23–187.88)	− 0.99 (− 1.08–0.89)	34.3 (27.12–43.96)	27.7 (22.07–35.37)	− 0.91 (− 0.98–0.84)	8.11 (4.81–12.71)	6.5 (3.86–10.22)	− 0.97 (-1.06–0.88)
Europe & Central Asia—WB	170.06 (128.06–226.95)	135.79 (102.15–183.85)	− 1.02 (− 1.12–0.93)	33.78 (26.73–43.26)	27.29 (21.7–34.77)	− 0.93 (− 1–0.86)	7.97 (4.73–12.47)	6.38 (3.79–10.01)	− 1 (− 1.1–0.91)
European Region	169.91 (127.94–226.8)	135.66 (102.02–183.74)	− 1.02 (− 1.11–0.92)	33.73 (26.69–43.2)	27.26 (21.68–34.74)	− 0.93 (-0.99–0.86)	7.97 (4.73–12.46)	6.38 (3.79–10.01)	− 1 (− 1.09–0.91)
High-income Asia Pacific	145.91 (107.08–200.81)	130.41 (94.96–180.97)	− 0.55 (− 0.66–0.44)	29.78 (23.73–38.99)	26.26 (20.75–34.69)	− 0.59 (− 0.69–0.5)	6.89 (4.05–11.03)	6.14 (3.59–10.01)	− 0.56 (− 0.66–0.45)
High-income North America	178.07 (130.94–238)	154.82 (115.59–206.09)	− 0.9 (-1.19–0.61)	41.12 (34.09–51.61)	38.28 (32.11–47.01)	− 0.56 (− 0.8–0.32)	8.65 (5.15–13.83)	7.64 (4.65–12.04)	− 0.82 (− 1.09–0.54)
Latin America & Caribbean—WB	138.72 (104.77–180.41)	118.39 (89.47–155.75)	− 0.18 (− 0.36–0)	29.62 (24–36.96)	25.72 (21.03–31.85)	− 0.12 (− 0.26–0.02)	6.61 (3.99–10.33)	5.66 (3.44–8.85)	− 0.17 (− 0.34–0.01)
Middle East & North Africa—WB	107.81 (83.22–138.57)	103.78 (75.44–146.19)	1.91 (1.22–2.61)	26.34 (18.61–41.88)	23.58 (16.81–35.05)	0.77 (0.32–1.23)	5.3 (3.38–8.07)	4.99 (2.98–8.07)	1.7 (1.05–2.36)
North Africa and Middle East	108.24 (81.88–147.43)	108.69 (76.81–161.3)	1.52 (0.9–2.13)	26.48 (18.03–43.22)	24.82 (17.01–38.3)	0.57 (0.19–0.96)	5.31 (3.34–8.25)	5.24 (3.08–8.58)	1.34 (0.77–1.91)
North America	178.04 (130.92–237.96)	154.81 (115.58–206.08)	− 0.9 (− 1.19–0.61)	41.11 (34.08–51.6)	38.27 (32.1–47)	− 0.56 (− 0.8–0.32)	8.64 (5.15–13.83)	7.64 (4.65–12.04)	− 0.81 (− 1.09–0.54)
Oceania	60.22 (46.56–77.9)	66.13 (50.37–86.16)	− 0.01 (− 0.61–0.6)	13.48 (11.08–16.6)	15.43 (12.7–19.04)	0.26 (− 0.1–0.63)	2.9 (1.78–4.48)	3.21 (1.99–4.94)	0.04 (− 0.52–0.61)
Region of the Americas	155.82 (116.92–204.74)	133.45 (101.2–175.33)	− 0.45 (− 0.57–0.32)	35.6 (29.5–44.24)	31.54 (26.33–38.7)	− 0.36 (− 0.46–0.25)	7.54 (4.53–11.75)	6.51 (3.94–10.2)	− 0.43 (− 0.55–0.31)
South–East Asia Region	99.09 (76.77–126.29)	91.74 (70.72–116.55)	− 0.31 (− 0.52–0.11)	23.26 (19.37–27.63)	23.01 (19.31–27.67)	− 0.09 (− 0.24–0.05)	4.82 (2.99–7.08)	4.54 (2.81–6.88)	− 0.26 (− 0.45–0.07)
South Asia	98.48 (76.1–125.13)	95.79 (73.83–122.92)	− 0.12 (− 0.31–0.08)	22.96 (19.15–27.5)	24.19 (20.4–28.99)	0.16 (0–0.31)	4.78 (2.97–7.11)	4.74 (2.94–7.18)	− 0.05 (− 0.24–0.14)
South Asia—WB	101.19 (78.51–129.72)	99.37 (75.91–127.3)	− 0.04 (− 0.23–0.14)	23.82 (19.74–29.08)	25.08 (21–30.06)	0.16 (0.02–0.29)	4.92 (3.05–7.24)	4.92 (3.05–7.3)	0.01 (− 0.16–0.18)
Southeast Asia	92.8 (71.14–120.41)	75.42 (58.22–95.92)	− 0.69 (− 1.01–0.37)	21.35 (17.58–26.01)	18.11 (14.99–22.03)	− 0.62 (− 0.8–0.45)	4.5 (2.79–6.73)	3.69 (2.28–5.65)	− 0.68 (− 0.97–0.39)
Southern Latin America	152.5 (112.01–212.65)	150.6 (110.15–208.48)	− 0.22 (− 0.3–0.13)	28.23 (21.68–37.9)	27.69 (21.21–37.21)	− 0.23 (− 0.3–0.16)	7.04 (4.04–11.44)	6.95 (4.03–11.26)	− 0.22 (− 0.3–0.14)
Southern Sub− Saharan Africa	97.4 (73.27–124.84)	80.11 (60.31–102.43)	− 0.51 (− 0.68–0.35)	25.32 (21.39–30.38)	20.64 (17.49–24.66)	− 0.44 (− 0.66–0.23)	4.87 (2.97–7.41)	4 (2.43–6.06)	− 0.49 (− 0.67–0.32)
Sub− Saharan Africa—WB	154.62 (90.87–283.64)	78.55 (59.5–101.71)	− 1.6 (− 2.2–0.99)	31.66 (22.76–46.6)	23.17 (18.98–28.95)	− 0.67 (− 0.97–0.37)	7.35 (3.91–13.88)	4.06 (2.56–6.02)	− 1.38 (− 1.92–0.85)
Tropical Latin America	139.2 (103.32–185.7)	120.92 (89.44–162.49)	− 0.44 (− 0.5–0.39)	31.75 (26.07–39.07)	27.77 (22.73–34.21)	− 0.4 (− 0.49–0.32)	6.73 (4.05–10.56)	5.87 (3.52–9.13)	− 0.43 (− 0.49–0.37)
Western Europe	122.36 (92.87–164.09)	108.26 (80.2–145.92)	− 0.63 (− 0.71–0.55)	24.23 (19.2–31.07)	21.47 (16.82–27.84)	− 0.63 (− 0.71–0.55)	5.74 (3.44–9.15)	5.08 (3–8.18)	− 0.63 (− 0.7–0.55)
Western pacific region	84.64 (64.5–112.5)	91.92 (68.82–120.42)	− 0.07 (− 0.34–0.2)	20.21 (16.91–24.64)	23.05 (19.47–27.77)	0.14 (− 0.11–0.39)	4.14 (2.55–6.31)	4.57 (2.76–6.97)	− 0.01 (− 0.27–0.25)
Western Sub− Saharan Africa	87.86 (64.38–118.08)	84.8 (62.91–111.95)	− 0.02 (− 0.31–0.28)	23.62 (19.98–28.05)	24.57 (20.96–28.94)	0.24 (0.11–0.36)	4.44 (2.69–6.64)	4.38 (2.7–6.57)	0.05 (− 0.2–0.3)
World bank high income	153.52 (114.72–205.55)	135.68 (101.35–181.43)	− 0.66 (− 0.8–0.52)	32.03 (25.86–41.01)	28.84 (23.28–36.75)	− 0.58 (− 0.7–0.46)	7.28 (4.31–11.56)	6.46 (3.84–10.31)	− 0.64 (− 0.78–0.51)
World bank low income	183.89 (97.66–365.77)	87.37 (64.54–119.42)	− 1.54 (− 2.38–0.7)	36.37 (23.24–62.01)	25.76 (19.29–38.08)	− 0.72 (− 1.13–0.32)	8.67 (4.35–17.62)	4.5 (2.8–6.88)	− 1.36 (− 2.1–0.61)
World bank lower middle income	99.84 (77.77–127.52)	92.5 (72.18–117.5)	− 0.13 (− 0.28–0.02)	23.18 (19.21–27.85)	22.99 (19.29–27.45)	0.01 (− 0.07–0.09)	4.85 (2.99–7.14)	4.56 (2.82–6.79)	− 0.09 (− 0.22–0.04)
World bank upper middle income	108.95 (83.6–139.83)	103.83 (78.22–135.38)	− 0.1 (− 0.24–0.03)	25.14 (20.77–30.57)	24.99 (20.87–30.34)	− 0.04 (− 0.17–0.09)	5.29 (3.21–8.16)	5.1 (3.1–7.85)	− 0.08 (− 0.21–0.05)

### Regional burden and trends of severe chest injury

**Fig. 1:**
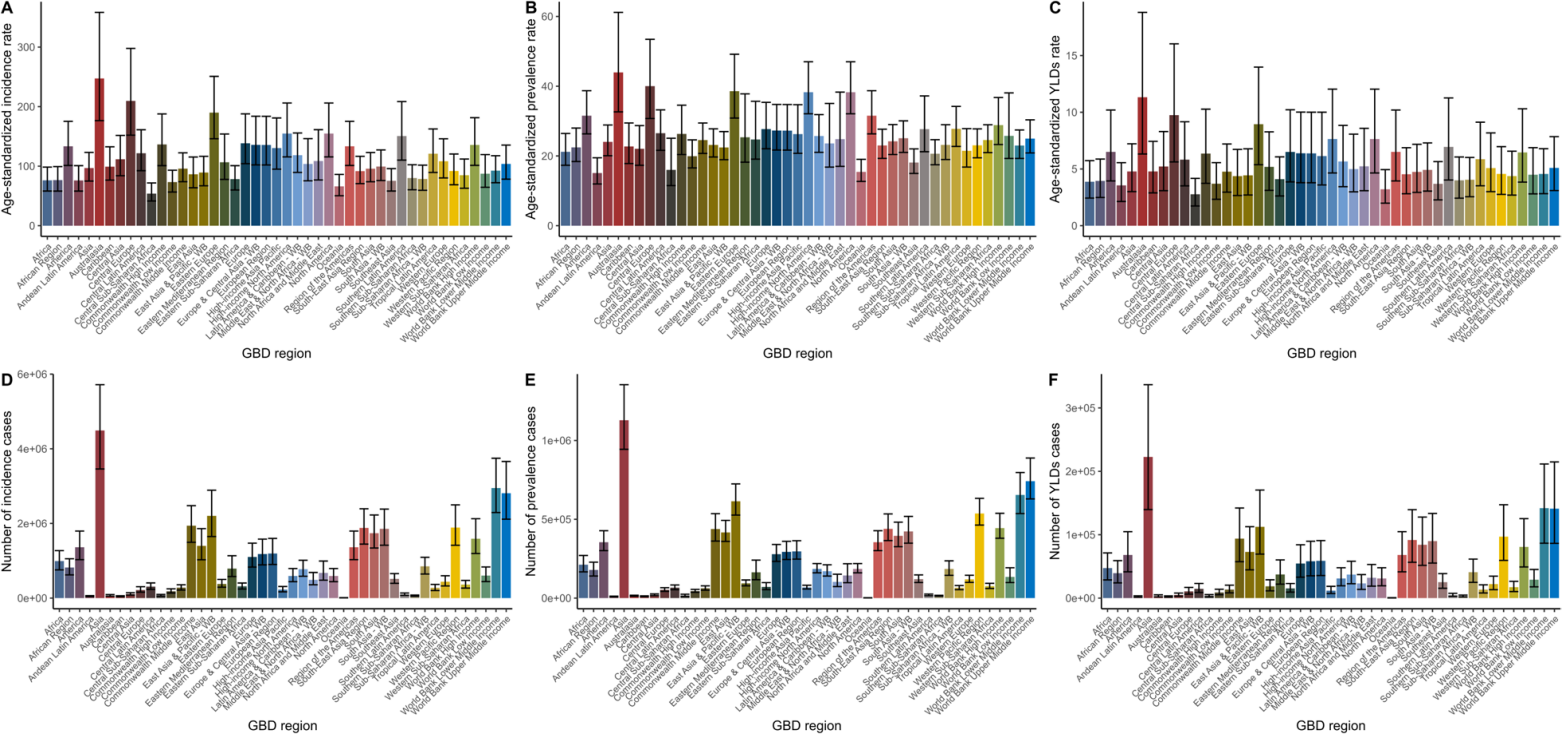
GBD regional distribution of ASIR, ASPR, and ASYR of severe chest injury in 2019. **A** ASIR. **B** ASPR. **C** ASYR. **D** the number of incidence cases. **E** the number of prevalent cases. **F** the number of YLD cases. *YLD* Years lived with disability, *ASIR* Age-standardized incidence rate, *ASPR* Age-standardized prevalence rate. *ASYR* Age-standardized YLDs rate

At the regional level, the ASIR of severe chest injury was highest in Australasia (247.36, 95% UI 176.45–358.3) and Central Europe (209.61, 95% UI 152.09–297.79). In contrast, Central Sub-Saharan Africa (53.99, 95% UI 41.48–71.82), Commonwealth low-income countries (73.28, 95% UI 56.56–93.24), and Oceania (66.13, 95% UI 50.37–86.16) exhibited the lowest ASIRs. The ASPR was highest in Australasia (43.94, 95% UI 32.64–61.17) and Central Europe (40.02, 95% UI 30.73–53.43), whereas Andean Latin America (15.1, 95% UI 11.97–19.47) and Oceania (15.43, 95% UI: 12.7–19.04) had the lowest rates. Similarly, the ASYR of severe chest injury was highest in Central Europe (9.74, 95% UI 5.62–16.03) and Eastern Europe (8.95, 95% UI 5.38–13.98). In contrast, Central Sub-Saharan Africa (2.77, 95% UI 1.74–4.17) and Oceania (3.21, 95% UI 1.99–4.94) had the lowest ASYRs (Fig. 1, Table 1). Furthermore, ASIR, ASPR, and ASYR demonstrated substantial increasing trends in Middle East and North Africa (WB), Eastern Mediterranean Region, and North Africa and Middle East, whereas considerably decreased trends were observed in Central Eastern Sub-Saharan Africa (Fig. S2, Table 1; Fig. 3, Table 1).

### National burden and trends of severe chest injury

**Fig. 2:**
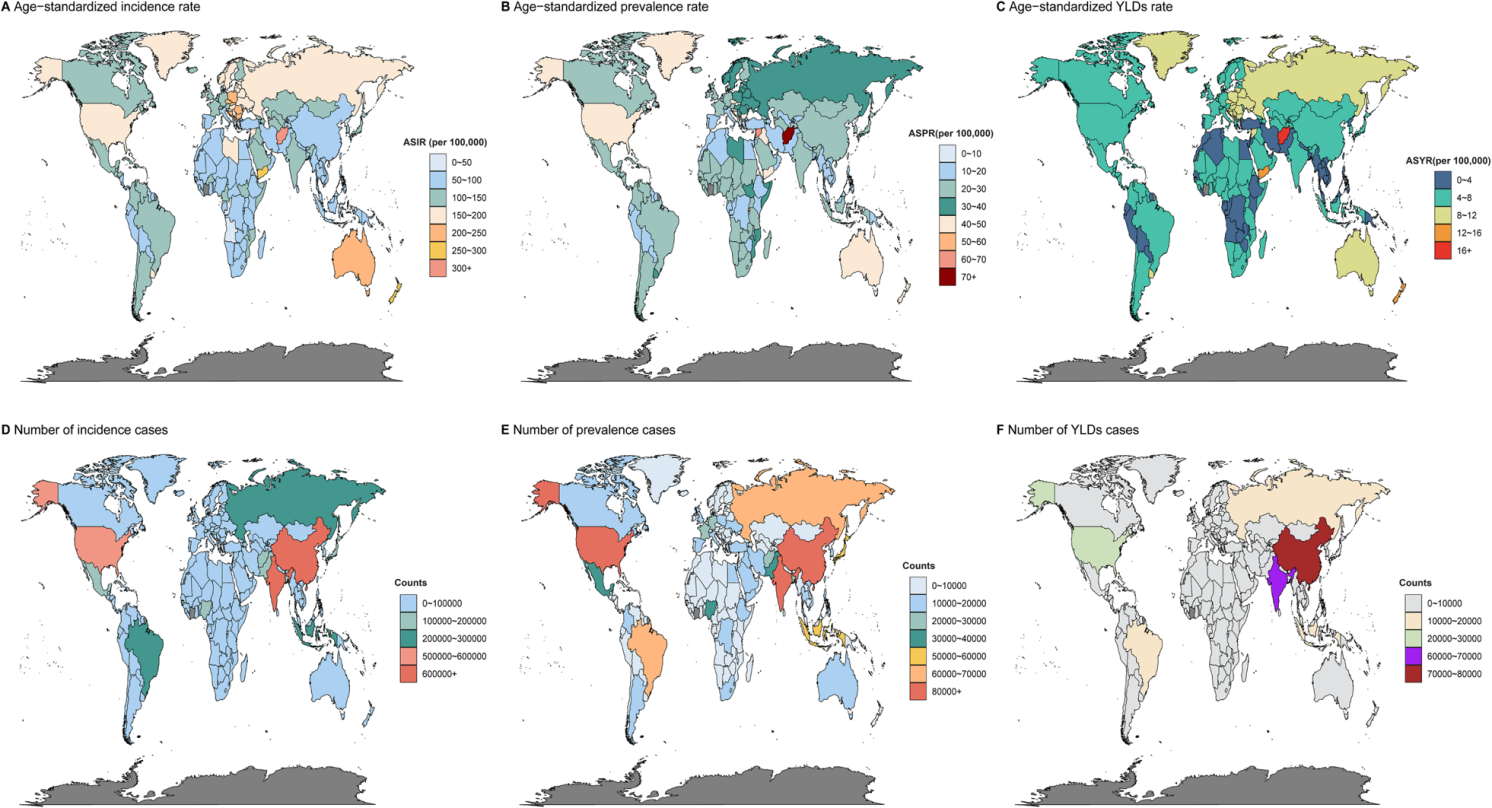
Geographical distribution of ASIR, ASPR, and ASYR of severe chest injury in 2019. **A** ASIR. **B** ASPR. **C** ASYR. **D** the number of incidence cases. **E** the number of prevalent cases. **F** the number of YLD cases. *YLD* Years lived with disability, *ASIR* Age-standardized incidence rate, *ASPR* Age-standardized prevalence rate, *ASYR* Age-standardized YLDs rate

**Fig. 3:**
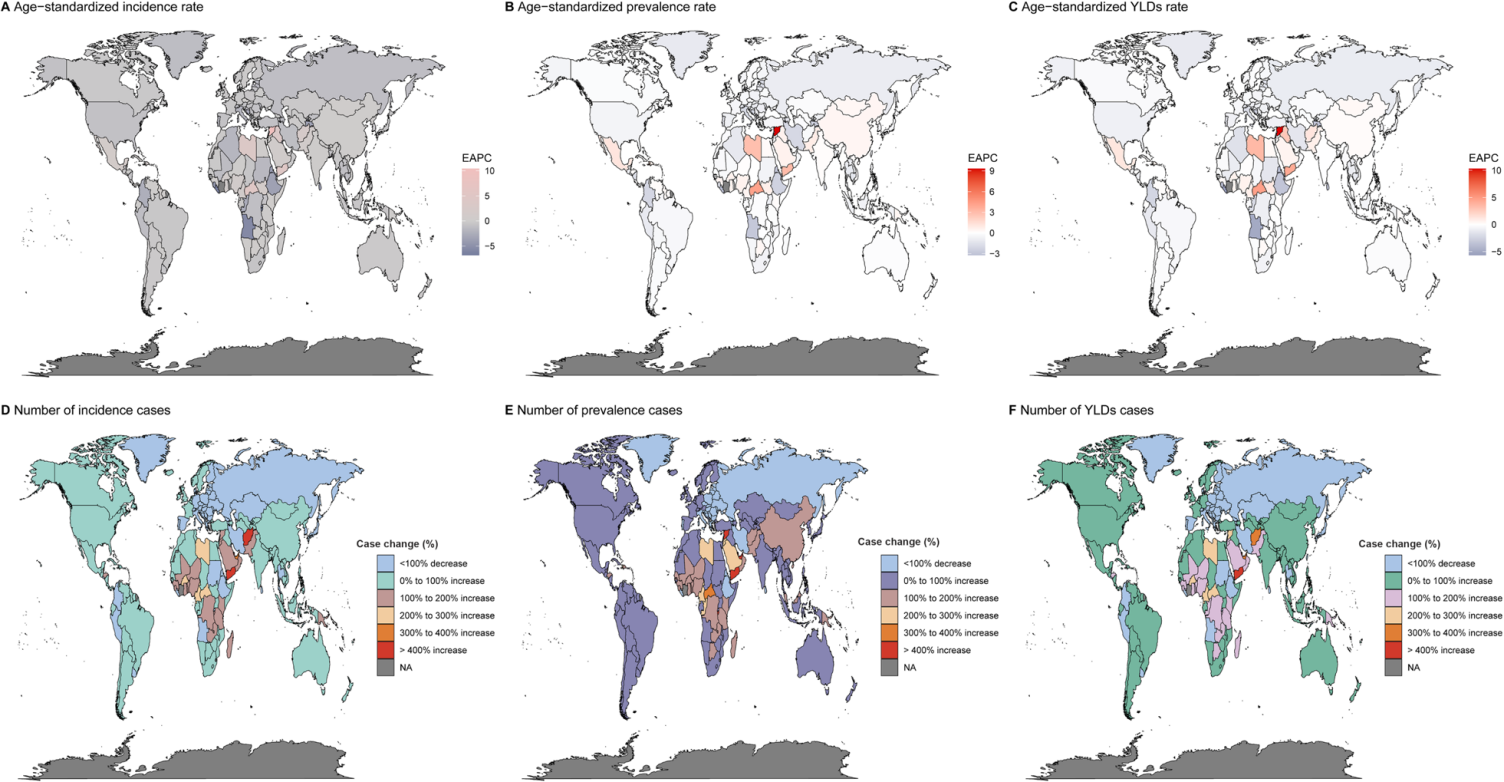
Geographical distribution of ASIR, ASPR, and ASYR of severe chest injury from 1990 to 2019. **A** EPACs in the ASIR. **B** EPACs in the ASPR. **C** EPACs in the ASYR. **D** the number of incidence cases. **E** the number of prevalent cases. **F** the number of YLD cases. *EAPCs* Estimated annual percentage changes. *YLD* Years lived with disability, *ASIR* Age-standardized incidence rate, *ASPR* Age-standardized prevalence rate. *ASYR* Age-standardized YLDs rate

In 2019, significant global health disparities were evident in the ASRs across countries. The ASIRs of Afghanistan (317.63, 95% UI 139.63–721.49), New Zealand (277.52, 95% UI 198.9–398.65), and Yemen (266.52, 95% UI 120.94–589.5) were among the highest, while the Democratic People’s Republic of Korea (41.02, 95% UI 31.74–52.69), Kiribati (42.76, 95% UI 33.03–54.86), and Taiwan (Province of China) (42.36, 95% UI 33.03–54.01) had the lowest ASIRs. In terms of ASPRs, Rwanda (52.38, 95% UI 29.11–119.96), the Syrian Arab Republic (66.29, 95% UI 27.01–157.35), and Afghanistan (89.25, 95% UI 34.39–222.24) reported the highest rates, significantly higher than the Democratic People’s Republic of Korea (9.26, 95% UI 7.6–11.45), Kiribati (9.62, 95% UI 7.91–11.84), and Taiwan (province of China) (9.86, 95% UI 8.19–12.01), which had the lowest ASPRs. Regarding ASYRs, Afghanistan (16.1, 95% UI 6.54–35.63), New Zealand (12.7, 95% UI 7.17–21.03), and Yemen (12.19, 95% UI 5.15–26.74) reported the highest rates, while the Democratic People’s Republic of Korea (1.99, 95% UI 1.22–3.03), Kiribati (2.06, 95% UI 1.27–3.15), and Taiwan (province of China) (2.07, 95% UI 1.26–3.18) had the lowest values (Fig. 2, Table S2). Besides, from 1990 to 2019, the Syrian Arab Republic, Central African Republic, and Yemen experienced the most significant increases in ASIR, ASPR, and ASYR, whereas Burundi, Timor-Leste, and Liberia showed the most notable declines in these metrics. These trends highlight substantial regional variations in severe chest injury rates (Fig. 3, Table S2).

### Social demographic index-specific trends of severe chest injury

The data analysis revealed no direct correlation between disease rates and socioeconomic development, as indicated by the SDI. While ASRs were similar across different SDI levels, regions with higher SDIs reported more incidence and prevalence cases in 2019, which may be attributed to the differences in health reporting systems or lifestyle-related risk factors. Additionally, the increase in long-duration cases in higher SDI regions may reflect better chronic disease management. These findings suggest a complex relationship between socioeconomic status and health outcomes (Fig. S3, Table S3).

**Fig. 4:**
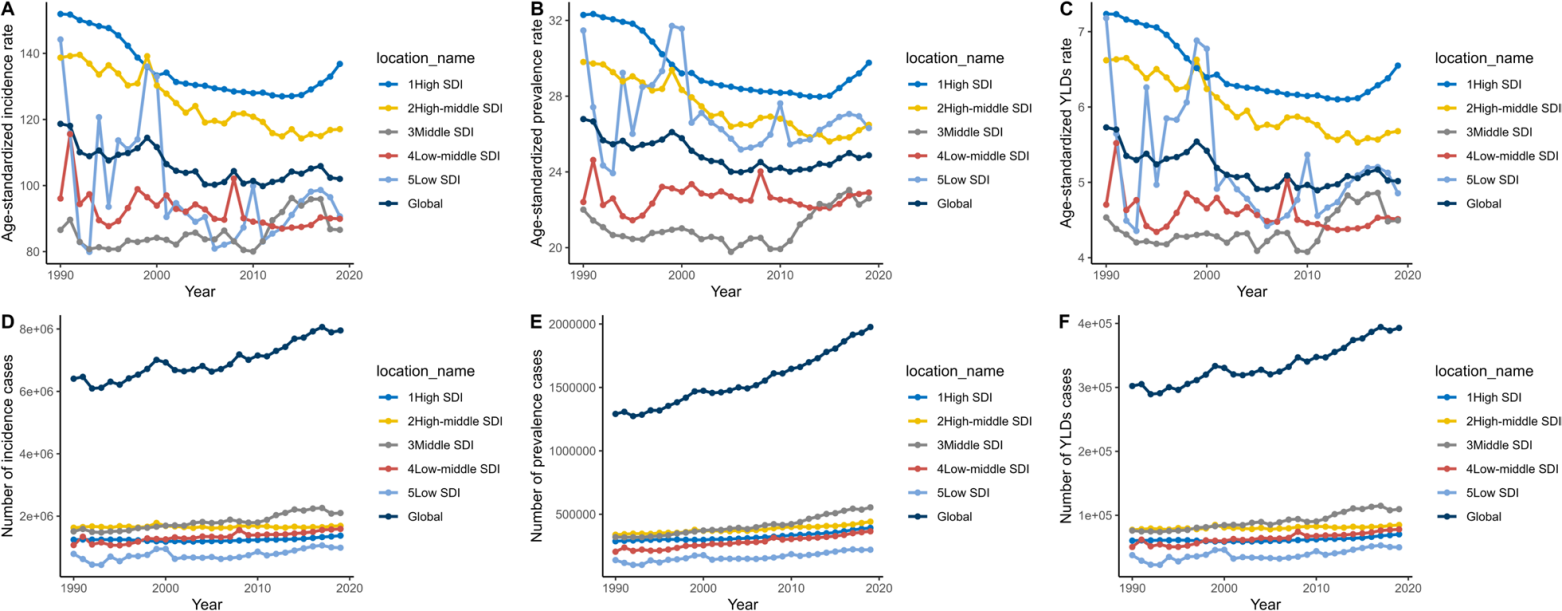
ASIR, ASPR, and ASYR of severe chest injury among SDI quintiles from 1990 to 2019. **A** EPACs in the ASIR. **B** EPACs in the ASPR. **C** EPACs in the ASYR. **D** the number of incidence cases. **E** the number of prevalent cases. **F** the number of YLD cases. *SDI* Social Demographic Index, *EAPCs* Estimated annual percentage changes. YLD = years lived with disability. ASIR = age-standardized incidence rate, *ASPR* Age-standardized prevalence rate. *ASYR* Age-standardized YLDs rate

The ASIR showed a noticeable decrease in high-, high-middle-, low-middle-, and low-SDI regions, with EPACs of − 0.57%, − 0.73%, − 0.37%, and − 0.8%, respectively. In contrast, the middle-SDI region experienced a slight increase in ASIR, with an EPAC of 0.35%. The global trend from 1990 to 2019 highlights the increasing disease burden across regions with varying socioeconomic statuses, with high-SDI regions showing a decrease in ASIR and low-SDI regions exhibiting fluctuating incidence rates. Prevalence rates and long-duration cases rose across all SDI categories, signaling a global challenge in managing chronic diseases (Fig. 4, Table S3).

### Severe chest injury burden and trends globally and in China

**Fig. 5:**
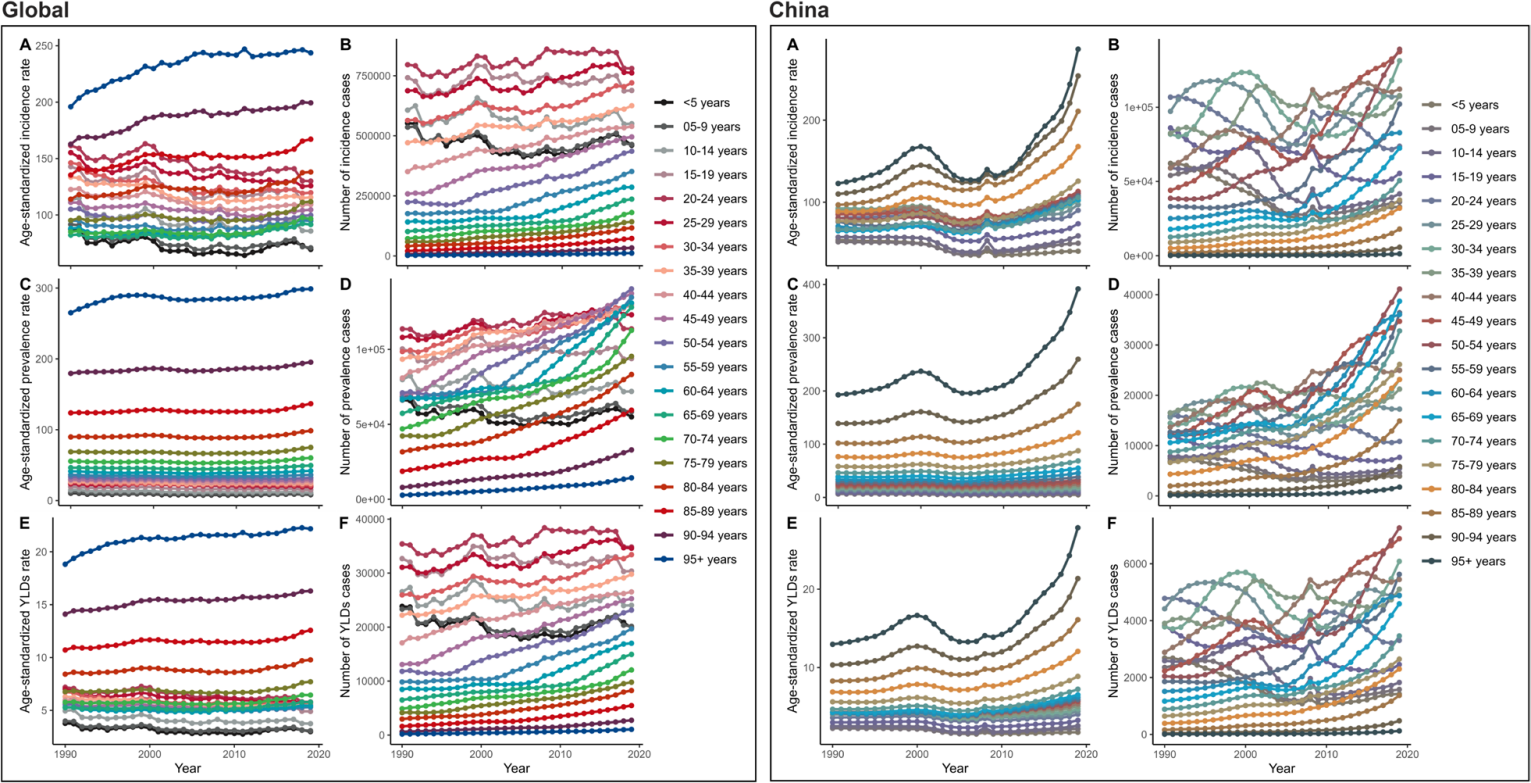
Age distribution of ASIR, ASPR, and ASYR of severe chest injury from 1990 to 2019 globally and in China. **A** ASIR. **B** ASPR. **C** ASYR. **D** the number of incidence cases. **E** the number of prevalent cases. **F** the number of YLD cases. *YLD* Years lived with disability, *ASIR* Age-standardized incidence rate, *ASPR* Age-standardized prevalence rate, *ASYR* Age-standardized YLDs rate

**Fig. 6:**
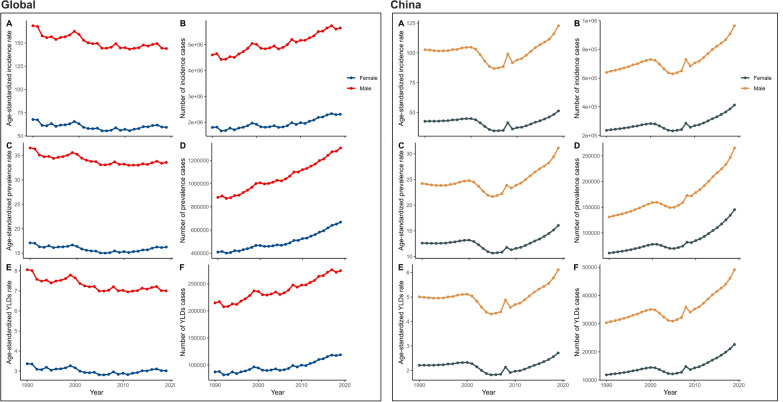
Gender distribution of ASIR, ASPR, and ASYR of severe chest injury from 1990 to 2019 globally and in China. **A** ASIR. **B** ASPR. **C** ASYR. **D** the number of incidence cases. **E** the number of prevalent cases. **F** the number of YLD cases. *YLD* Years lived with disability, *ASIR* Age-standardized incidence rate, *ASPR* Age-standardized prevalence rate, *ASYR* Age-standardized YLDs rate

**Fig. 7:**
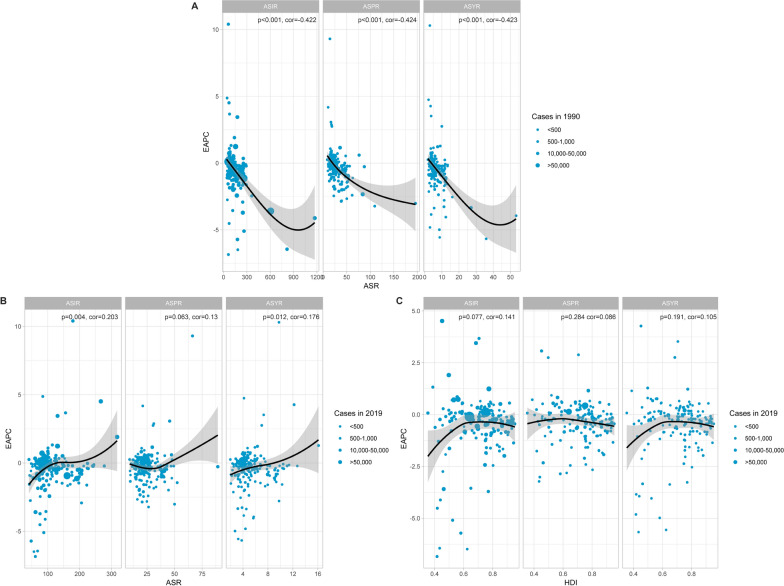
Influential factors affecting EAPC in the GBD. **A** correlation of EAPC with ASIR, ASPR, and ASYR in 1990. **B** correlation of EAPC with ASIR, ASPR, and ASYR in 2019. **C** correlation of EAPC with HDI in 2019. r and P values were obtained using Pearson correlation. *EAPCs* Estimated annual percentage changes, *YLD* Years lived with disability, *ASIR* Age-standardized incidence rate, *ASPR* Age-standardized prevalence rate, *ASYR* Age-standardized YLDs rate

Globally, the number of new severe chest injury cases has remained relatively stable from 1990 to 2019, with the ASIR decreasing slightly from 118.66 (95% UI 90.89–154.47) to 102 (95% UI 78.66–130.57), indicating effective control of new cases relative to population size (EAPC: − 0.41%, 95% CI: − 0.54, − 0.28). Similarly, the ASPR showed a declining trend from 26.79 (95% UI 22.08–32.61) to 24.88 (95% UI 20.79–29.88), with an EAPC of − 0.26% (95% CI − 0.35, − 0.17). In contrast, China experienced increasing trends in ASIR, ASPR, and ASYR, with respective EAPCs of 0.11% (95% CI − 0.25, 0.47), 0.43% (95% CI 0.11–0.76), and 0.21% (95% CI − 0.14, 0.56). The rise in these rates suggests a growing challenge in managing both new and existing cases, emphasizing the need for enhanced healthcare infrastructure and policies (Fig. S4). In 2019, globally, the ASIR, ASPR, and ASYR increased with age, peaking at age 95 and above. Incidence and YLD peaked in the 20–34 age group, with a normal distribution pattern peaking in the 45–54 age group. In China, these rates also increased with age, with peak incidence in the 45–54 age group and YLD following a similar pattern (Fig. S5, Table S4). Both globally and in China, the ASIR and ASPR increased with age, with notable peaks in the oldest age cohorts and significant case numbers in younger age groups, reflecting the broad impact of disability (Fig. 5, Table S4). When comparing global trends to those in China, distinct patterns emerge. Globally, the ASIR, ASPR, and ASYR remained stable over time for both genders, with females generally having higher rates. However, in China, a pronounced surge in these rates occurred around the mid-2000s, particularly among males, indicating a rapidly escalating disease burden and highlighting the need for targeted interventions (Figs. 6 and S6, Table S5). To understand the GBD in severe chest injury, factors influencing the EAPC, including the ASIR, ASPR, and ASYR, were analyzed along with the HDI. A strong negative correlation between EAPC and ASIR, ASPR, and ASYR in 1990 (r =  − 0.422, − 0.423, and − 0.243; all p < 0.001) weakened by 2019, with slight positive correlations for ASIR and ASYR (r = 0.202, p = 0.003; r = 0.175, p = 0.011). No significant correlation with ASPR was found (r = 0.130, p = 0.063) nor between EPAC and HDI (Fig. 7).

### Causes of severe chest injury

**Fig. 8:**
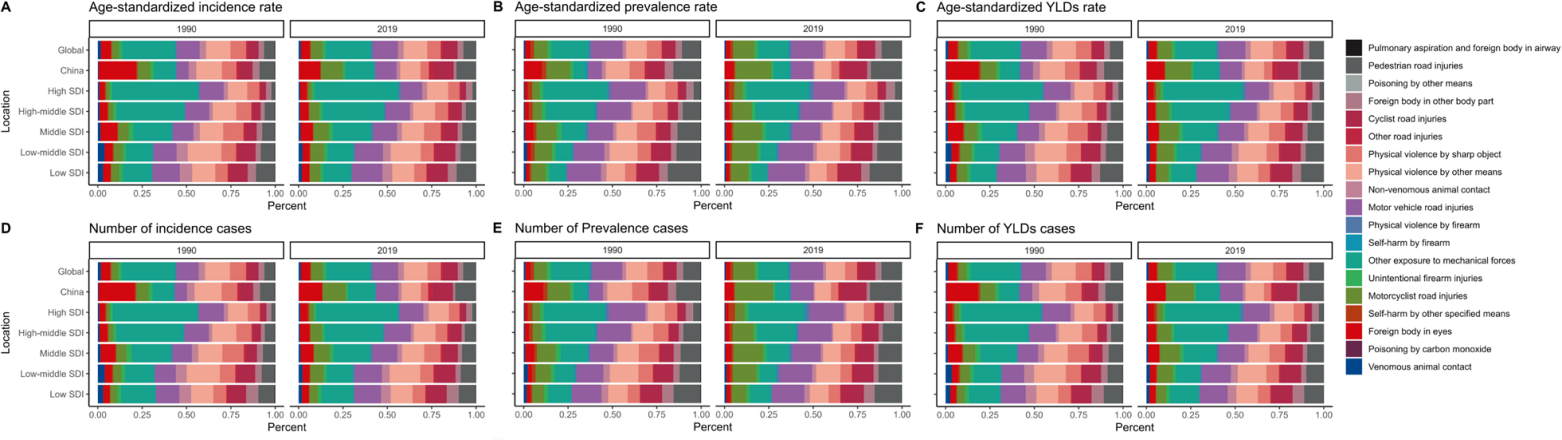
Level 4 causes of severe chest injury in various SDI regions and globally versus China. **A** ASIR. **B** ASPR. **C** ASYR. **D** the number of incidence cases. **E** the number of prevalent cases. **F** the number of YLD cases. *YLD* Years lived with disability, *ASIR* Age-standardized incidence rate, *ASPR* Age-standardized prevalence rate, *ASYR* Age-standardized YLDs rate, *SDI* Social Demographic Index

The GBD study presents a comprehensive framework for analyzing the global landscape of morbidity and mortality from 1990 to 2019, organized into four hierarchical levels for detailed cause analysis. This framework enables a detailed examination of causes ranging from broad categories like communicable diseases, noncommunicable diseases, and injuries at Level 1 to highly specific conditions at Level 4. This study employed the SDI to assess changes in injury metrics globally, revealing decreasing trends in ASIR, ASPR, and ASYR, particularly in high-SDI regions. Conversely, low-SDI regions experienced an increase. Despite these trends, global incidences, prevalence cases, and YLD have shown an upward trajectory, mainly concentrated in low-SDI areas. Analysis at Level 2 demonstrated shifts in injury metrics globally, with improvements in high-SDI regions contrasted by challenges faced in low-SDI regions. China mirrors these trends with reductions in rates but a rise in absolute injury cases (Fig. S7, Table S6). At Level 3, the study highlights “falls” and “road injuries” as enduring global health challenges, including China (Fig. S8, Table S6). At Level 4, the study identified falls, road injuries, and interpersonal violence as the top global causes of injuries in 2019, with a specific mention of poisoning in China, indicating unique regional health challenges. This detailed analysis highlights shifts in the epidemiological landscape from 1990 to 2019, offering insights into evolving public health priorities and the necessity for targeted intervention strategies (Fig. 8, Table S7).

**Fig. 9:**
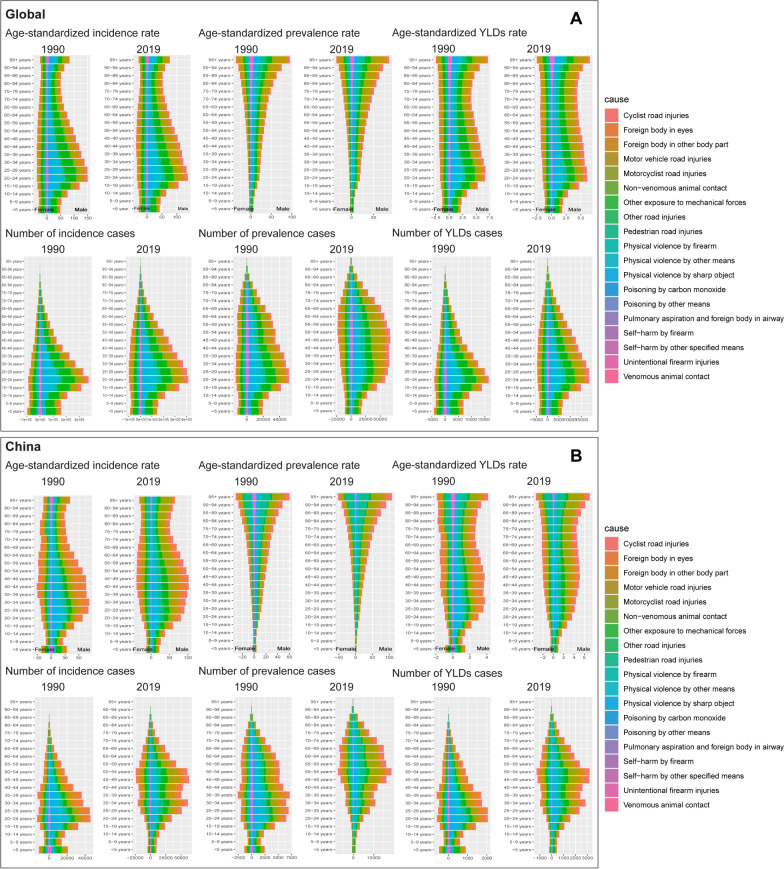
Level 4 causes of severe chest injury globally and in China, stratified by age group and gender, between 1990 and 2019. **A** global stacked pyramid charts of the ASIR, ASPR, and ASYR along with the number of incidence, prevalence, and YLD. **B** China stacked pyramid charts of the ASIR, ASPR, and ASYR along with the number of incidence, prevalence, and YLD. *YLD* Years lived with disability, *ASIR* Age-standardized incidence rate, *ASPR* Age-standardized prevalence rate, *ASYR* Age-standardized YLDs rate, *SDI* Social Demographic Index

On the global Level 4, young adults (around 20–30 years old) were the most affected age group by severe chest injuries in 1990, primarily due to road injuries, notably motor vehicle accidents. By 2019, while the peak burden still fell on young adults, there was a notable increase in the middle-aged population experiencing severe chest injuries. The incidence and prevalence of chest injuries from road accidents showed a decrease. Notably, self-harm from firearms and sharp objects emerged as a more prominent cause of chest injuries over time. In China, motor vehicle road injuries were a major cause of chest injuries in 1990, with decreasing incidence rates observed by 2019. The distribution of causes became more diversified in 2019 than in 1990, and the age groups affected by severe chest injuries broadened, with a notable burden seen in the 40–60-year-old age bracket (Fig. 9, Tables S8 and S9). For further detailed information on Level 2 and Level 3 analyses, please refer to Figure S9, Tables S12 and S13, Figure S10, and Tables S10 and S11.

### Age, period, and cohort analysis of the ASIR, ASPR, and ASYR in severe chest injury globally and in China

**Fig. 10:**
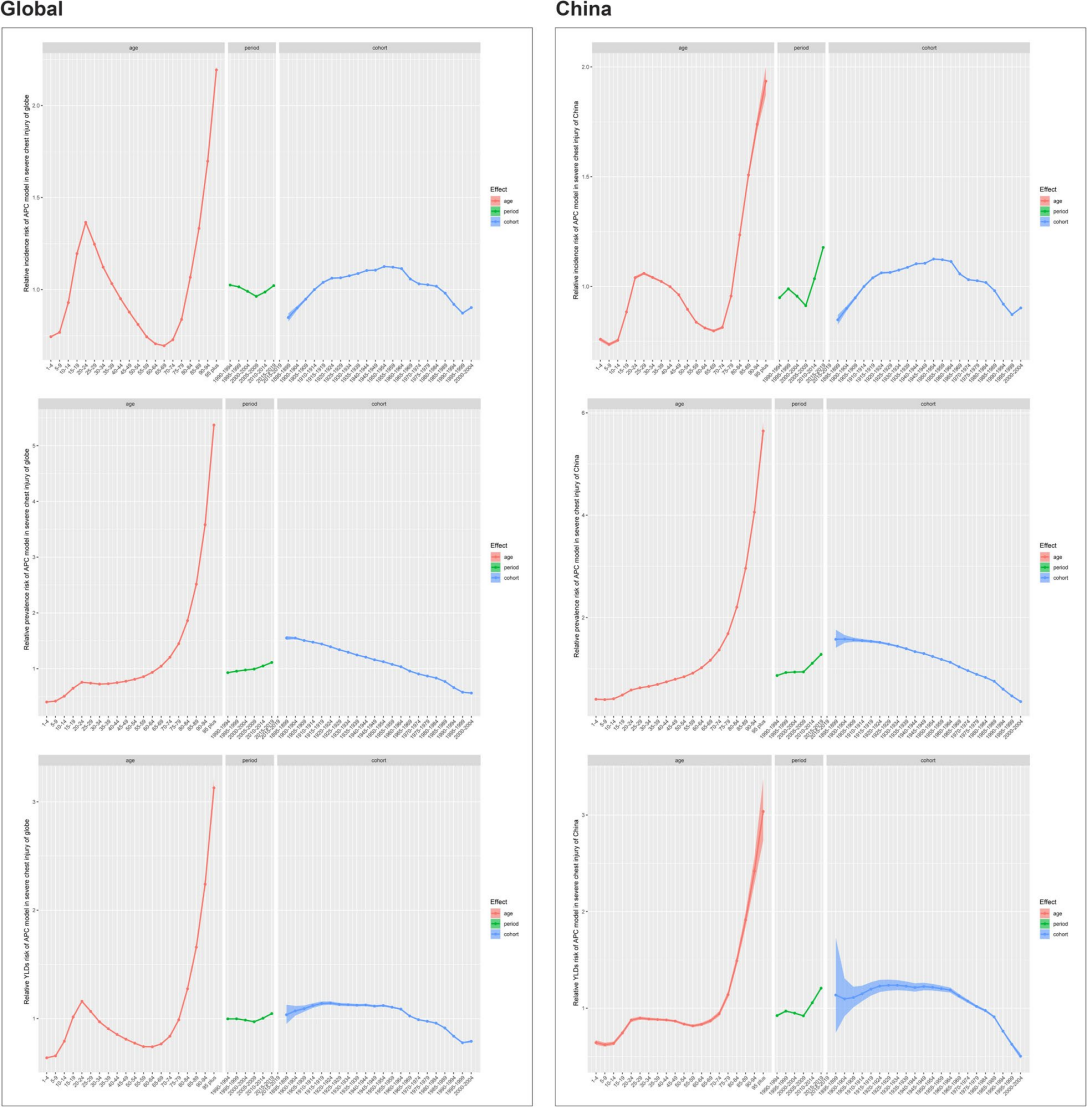
Age, period, and cohort effect relative risk of severe chest injury incidence, prevalence, and YLD globally and in China, 1990–2019. **A** ASIR. **B** ASPR. **C** ASYR. **D** ASIR. **E** ASPR. **F** ASYR. *YLD* Years lived with disability, *ASIR* Age-standardized incidence rate, *ASPR* Age-standardized prevalence rate, *ASYR* Age-standardized YLDs rate

The APC-IE method provided coefficients for age, period, and cohort effects, which were transformed into exponential values (exp(coef.) = e coef.), representing the relative risk (RR) of a specific age, period, or birth cohort compared to the average level [25, 26]. For the global analysis, after adjusting for period and cohort influences, incidence rate’s age effect exhibited a minor peak at ages 20–24 (RR = 1.36), followed by a gradual decline, reaching its lowest at ages 65–69 (RR = 0.69), before increasing again. Period effect on incidence, after controlling for cohort and age effects, exhibited a slight decrease from 1990–1994 (RR = 1.02) to 2005–2009 (RR = 0.96), followed by an increase up to 2019 (RR = 1.02). Moreover, the cohort effect indicated a steady rise in incidence risk from 1895–1899 (RR = 0.84) to a peak in 1950–1954 (RR = 1.12), followed by a decrease to 2000–2004 (RR = 0.90) in birth cohorts. In China, age effect on incidence rate’s RR peaked at ages 25–29 (RR = 1.05), then gradually declined, reaching its lowest at 65–69 (RR = 0.79), before rising again. The period effect showed a slight increase from 1990 to 1999 (RR = 0.94) and a dip in 2005–2009 (RR = 0.91), followed by a gradual rise through 2019 (RR = 1.17). Similarly, cohort effect in China increased from 1895–1899 (RR = 0.84) to 1950–1954 (RR = 1.12), with a subsequent decline in 2000–2004 (RR = 0.90). These trends were consistent across both ASPR and ASYR (Figs. 10 and S2, Table S14).

### The ARIMA model can predict the severe chest injury trend from 2020 to 2050 globally and in China

**Fig. 11:**
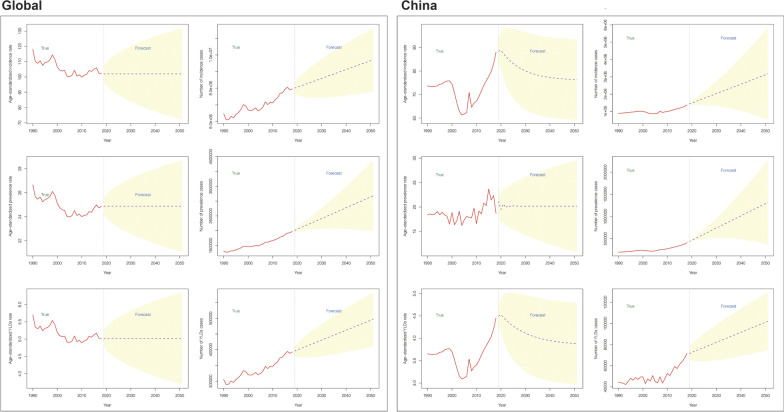
Predicted trends of severe chest injury in 2020–2050 by the ARIMA model. **A** Global ARIMA model. **B** China ARIMA model. *ARIMA* Autoregressive integrated moving average

The estimated numbers for incidence, prevalence, and YLD, along with the ASIR, ASPR, and ASYR for severe chest injury, were analyzed. According to the ARIMA predictive models, a consistent trend is forecasted globally from 2020 to 2050. ASIR, ASPR, and ASYR demonstrate no significant deviations in trend from 2020 onward, with the numbers continuing to rise steadily. In the context of China, the ARIMA model suggests a continuous upward trend in the incidence, prevalence, and YLD of severe chest injury. However, the ASIR and ASYR have been decreasing since 2020, while the ASPR shows an upward trend (Fig. 11, Table S15).

## Discussion

Thoracic trauma accounts for 10%–15% of all trauma admissions and contributes to 25% of traumatic deaths [27, 28]. Severe thoracic injuries have a high baseline mortality rate [29] and frequently lead to pulmonary complications, such as pneumothorax, hemothorax, pulmonary contusions, and atelectasis, increasing the risk of ventilator-dependent respiratory failure [30]. In addition, thoracic trauma is the third leading cause of death among multitrauma patients and is linked to poor short-term outcomes, responsible for up to 25% of trauma-related fatalities [31, 33]. This study is the first, to our knowledge, to delineate the incidence, prevalence, and YLD associated with severe chest injury by analyzing the GBD database. severe chest injury has a substantial impact on patient outcomes, often necessitating immediate and specialized care [27]. These injuries can encompass rib fractures, pulmonary contusions, and pneumothorax, necessitating interventions such as surgical stabilization of rib fractures within the initial 72 h to enhance recovery [28]. Technological advancements in extracorporeal lung support, such as pump-less extracorporeal lung assist and veno-venous ECMO, have shown promise in managing severe respiratory failure [29]. This highlights the significance of timely and specialized interventions to reduce morbidity and mortality associated with severe chest injury.

The GBD 2019 study provided deep insights into the global and regional burden of severe chest injury, revealing trends over time with significant epidemiological implications. Although ASIR, ASPR, and ASYR declined globally, the absolute number of cases increased due to population growth [17, 30, 31]. This suggests progress in prevention, early diagnosis, and management, but also highlights the ongoing challenge of a growing burden due to demographic shifts. Regional disparities in severe chest injury burden exist and correlate closely with socioeconomic development, healthcare access, and the prevalence of risk factors like road traffic accidents and occupational hazards [30, 31]. China, for instance, exhibits unique trends in long-term severe chest injury cases stemming from its aging populations, rapid urbanization, and industrial accidents. The higher incidence among males and the increasing trend with age emphasize the need for targeted interventions. This highlights the importance of improved emergency care, chronic disease management for those living with severe chest injury, and initiatives to reduce known risk factors. Furthermore, enhanced research and robust data collection are crucial for informing effective prevention and management strategies. Global collaboration in severe chest injury research and prevention, through the sharing of best practices and resource pooling, is vital to reducing the burden of severe chest injury, particularly in low- and middle-income countries. Recognizing limitations in current data highlights the need for future longitudinal studies and rigorous intervention assessments [30].

The GBD’s hierarchical framework, moving from general injury categories to specific causes, guides targeted public health interventions. Globally, injury rates have decreased, particularly in high-SDI regions [31]. Advancements in healthcare, safety regulations, and public health initiatives have contributed to this decline. However, low-SDI regions face a growing public health challenge with rising injury rates. Notably, high-SDI regions have seen a decrease in transport-related injuries, likely due to improved road safety measures. Variations in the rates of self-harm, interpersonal violence, and unintentional injuries across different SDI regions further emphasize the need for region-specific approaches to injury prevention [32]. Falls and road injuries are the leading causes of injuries globally and in China [33], highlighting the need for ongoing interventions to prevent falls and enhance road safety. The prevalence of poisoning by other means in China points to unique cultural, environmental, and socioeconomic factors [34] that need to be addressed in public health strategies. Effectively addressing these issues requires a comprehensive understanding of the complex nature of injury causes and their impacts across regions, necessitating targeted interventions, increased health service funding, and ongoing monitoring of injury trends [35]. In China, there has been a notable decrease in road injuries among young people due to improved traffic regulation. However, there has been an increase in falls among the elderly and self-harm-related injuries, presenting new public health challenges. These ongoing injury burdens emphasize the need for implementing targeted prevention strategies and strengthening healthcare systems to manage long-term disability outcomes. Adaptive policies are crucial to addressing both acute care and rehabilitation for injured patients.

This study employs the APC-IE model to analyze severe chest injury trends globally and in China, highlighting variations in ASIR, ASPR, and ASYR. Globally, a slight peak in severe chest injury risk emerges among young adults aged 20–24, followed by a decline with age, indicating higher engagement in risky activities in this age group. In contrast, China shows a minor peak at ages 25–29, potentially reflecting lower risk among Chinese youth due to safety improvements and public health initiatives. Period effects globally show an initial decrease in risk over time, followed by an increase, reflecting health advancements and socioeconomic changes. In China, severe chest injury risk increased from 1990 to 1999, decreased until 2009, and then rose again, possibly due to a combination of improved safety measures and healthcare enhancements [36]. Finally, cohort effects reveal fluctuating risks across different birth cohorts, suggesting the enduring impact of societal and lifestyle trends on injury patterns.

Global ARIMA forecasts show stable ASIR, ASPR, and ASYR despite rising severe chest injury cases, reflecting historical trends and improved healthcare. In China, ARIMA predicts growing severe chest injury rates despite declining ASIR and ASYR since 2020, possibly due to aging or improved diagnostics. These forecasts emphasize the need for ongoing and targeted public health strategies. Globally, the focus should be on stabilizing and ultimately reducing the absolute number of severe chest injuries, even if ASRs remain steady. In China, priority should be given to addressing the specific factors driving the increase in severe chest injury incidence and prevalence, with a particular emphasis on enhancing severe chest injury prevention, early detection, and management capabilities. Furthermore, these findings highlight the importance of using ARIMA and other forecasting models for healthcare planning and resource allocation. By predicting future trends, healthcare systems can better prepare to address the evolving burden of severe chest injury, ensuring timely and effective interventions.

## Conclusions

This study provides a groundbreaking analysis of severe chest injury data from the GBD 2019 study database, offering valuable insights into severe chest injury incidence, prevalence, and YLD. While progress has been made in reducing ASRs of severe chest injury, challenges persist due to demographic shifts resulting in an increase in the absolute number of patients. Advances in medical technology and care practices hold promise for severe chest injury management, but this study highlights the critical need for timely intervention and specialized care. Identified regional disparities in severe chest injury burden are influenced by factors such as socioeconomic status, healthcare access, and risk exposure. The findings emphasize the need for targeted interventions, improved healthcare strategies, and global collaboration to mitigate the impact of severe chest injury, particularly in regions facing significant challenges.

### Limitations of the study

Despite its impact, this study has certain limitations. First, critics point to its heavy reliance on disease modeling and indirect estimates, particularly for regions with limited health data, possibly resulting in inaccuracies. The methodology involving ASIR, ASPR, and ASYR has been criticized for its weighting of disabilities and diseases, raising concerns about inherent value judgments in these choices. Furthermore, complexity and opacity of the GBD models may hinder their accessibility and utility for policymakers and local health officials seeking practical insights. Moreover, although this study provides a comprehensive analysis of severe chest injuries globally and in China, it lacks detailed and country-specific data. Analysis using public health resources such as the GBD is necessary for further elucidation. A substantial limitation of this research is its failure to examine the situation in each individual country. Lastly, this study exclusively focused on data pertaining to nonfatal severe chest injuries, lacking further support from indicators such as mortality and disability-adjusted life years. These gaps should be addressed in future studies.

## Supplementary Information

Additional file 1.

Additional file 2.

## Data Availability

The datasets generated during this study are available in the Global Health Data Exchange query tool (http://ghdx.healthdata.org/gbd‐resul ts‐tool).

## Abbreviations

GBD: Global Burden of Disease
ASR: Age-standardized rate
ASIR: Age‐standardized incidence rate
ASPR: Age-standardized prevalence rate
ASYR: Age-standardized lived with disability rate
IE: Intrinsic estimator
ARIMA: Autoregressive integrated moving average
APC-IE: Age-Period-Cohort Intrinsic Estimator
HDI: Human development index
UI: Uncertainty interval
CI: Confidence interval
EAPC: Estimated annual percentage changes
ICD: International classification of diseases
SDI: Sociodemographic index
EC MO: Extracorporeal membrane oxygenation
