# Supplementary figures and images for: Insights into epidemiological trends of severe chest injuries: an analysis of age, period, and cohort from 1990 to 2019 using the Global Burden of Disease study 2019
Source: Scand J Trauma Resusc Emerg Med. 2024 Sep 16;32:89. doi: 10.1186/s13049-024-01258-2 (PMC11403847; doi:10.1186/s13049-024-01258-2)

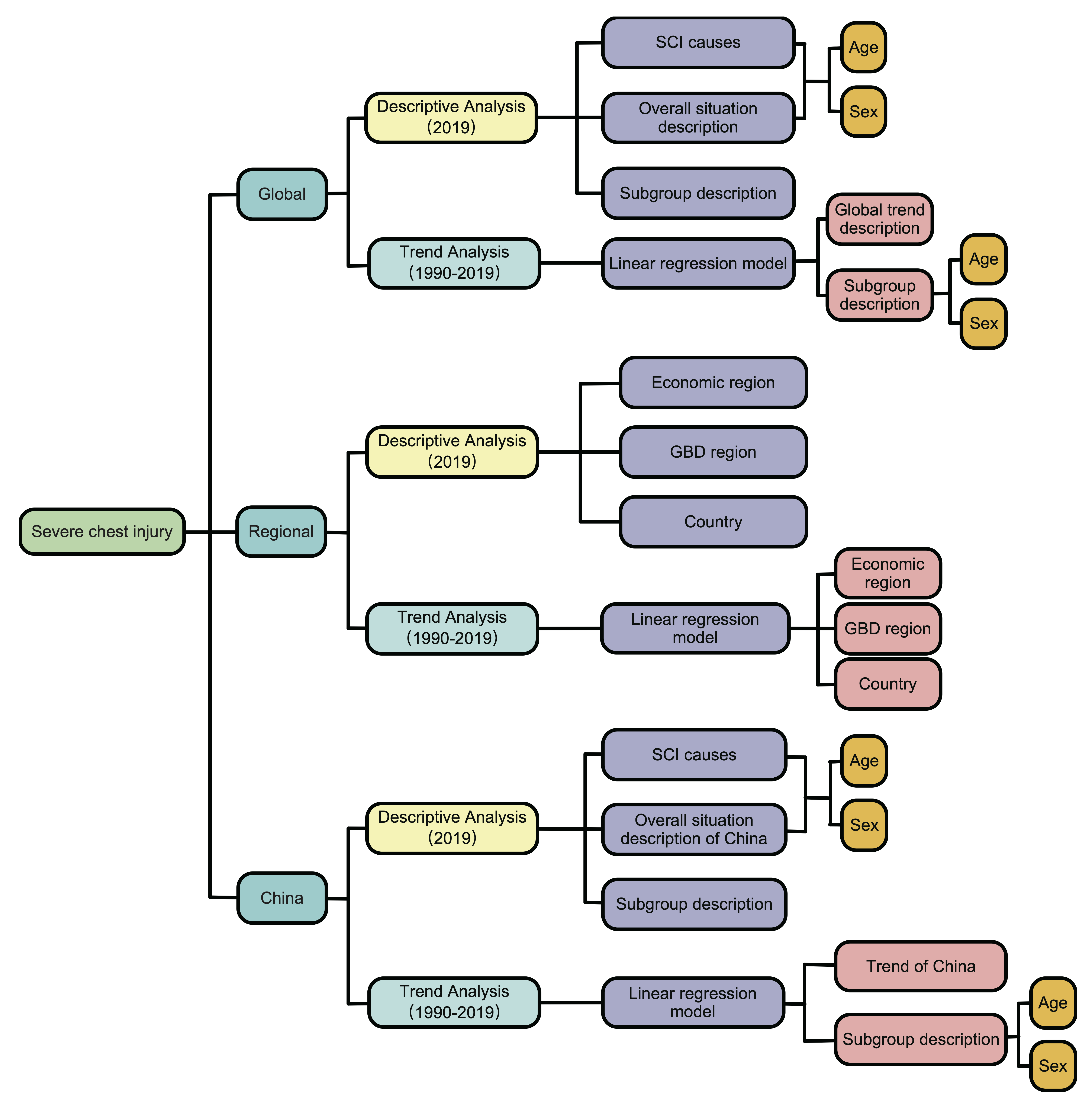

Supplement: Supplementary file 2 — Additional file 2. [file 13049_2024_1258_MOESM2_ESM.zip › additional file 2/Additional file 2 Fig. S1.png]

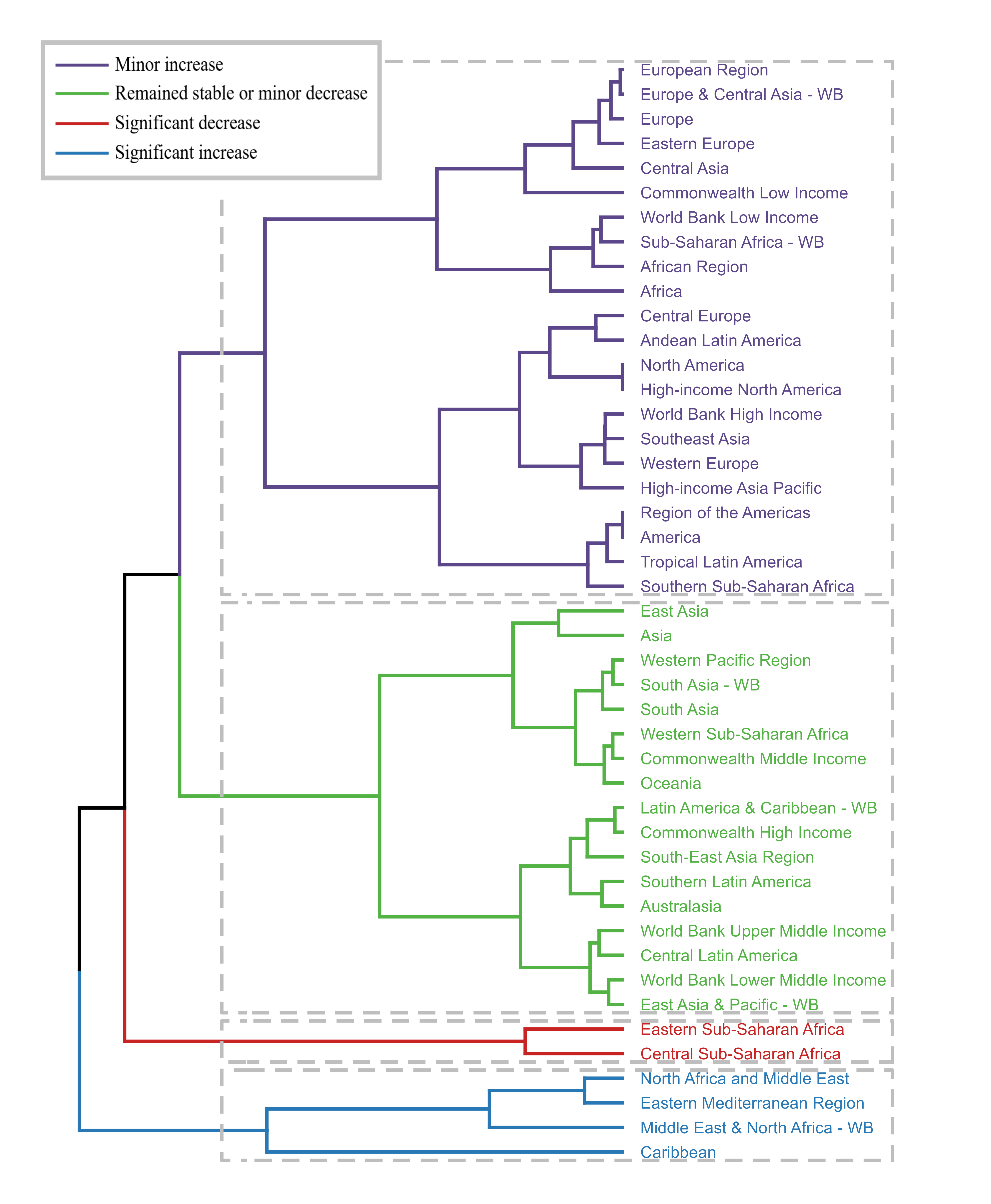

Supplement: Supplementary file 2 — Additional file 2. [file 13049_2024_1258_MOESM2_ESM.zip › additional file 2/Additional file 2 Fig. S2.png]

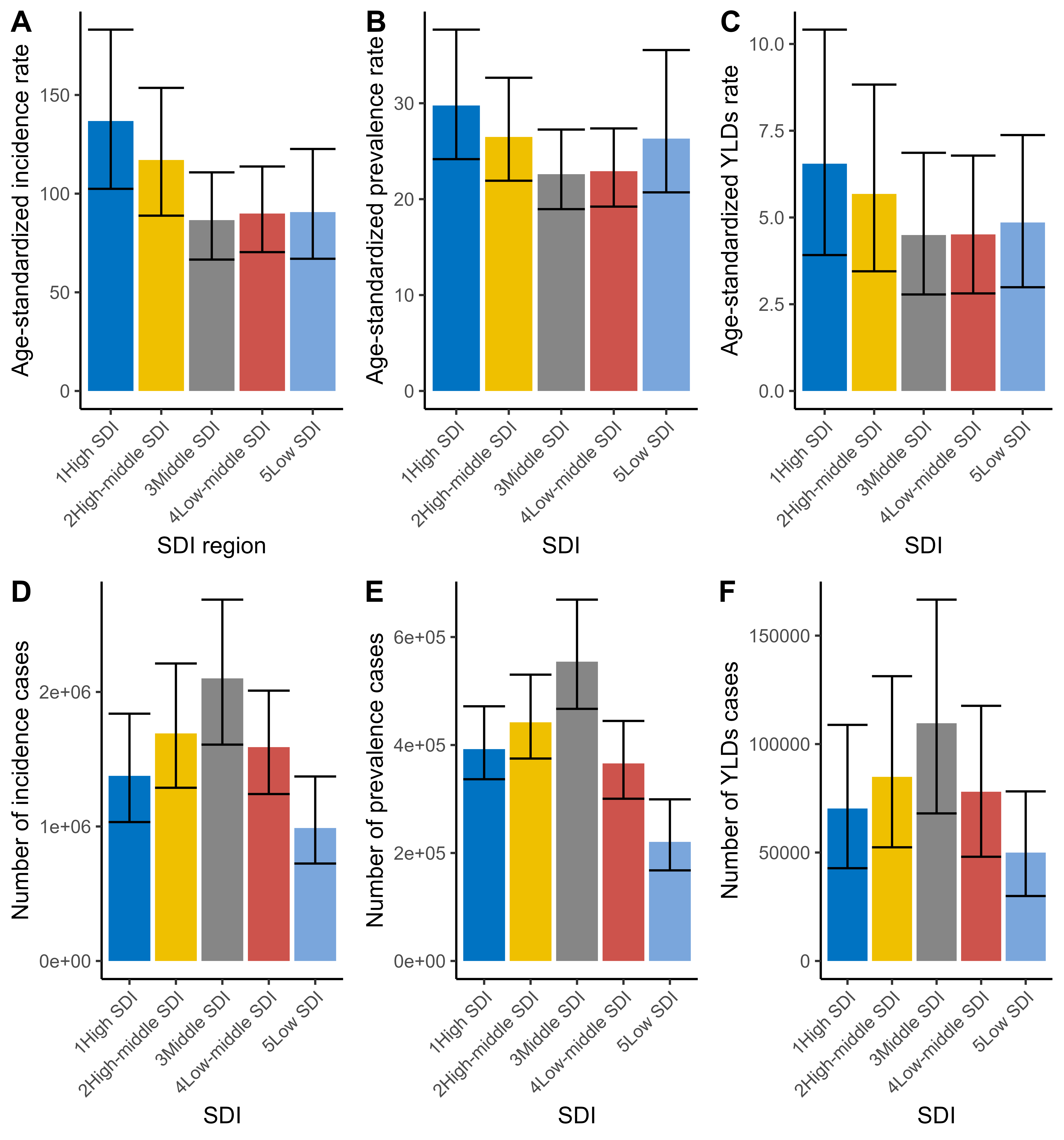

Supplement: Supplementary file 2 — Additional file 2. [file 13049_2024_1258_MOESM2_ESM.zip › additional file 2/Additional file 2 Fig. S3.png]

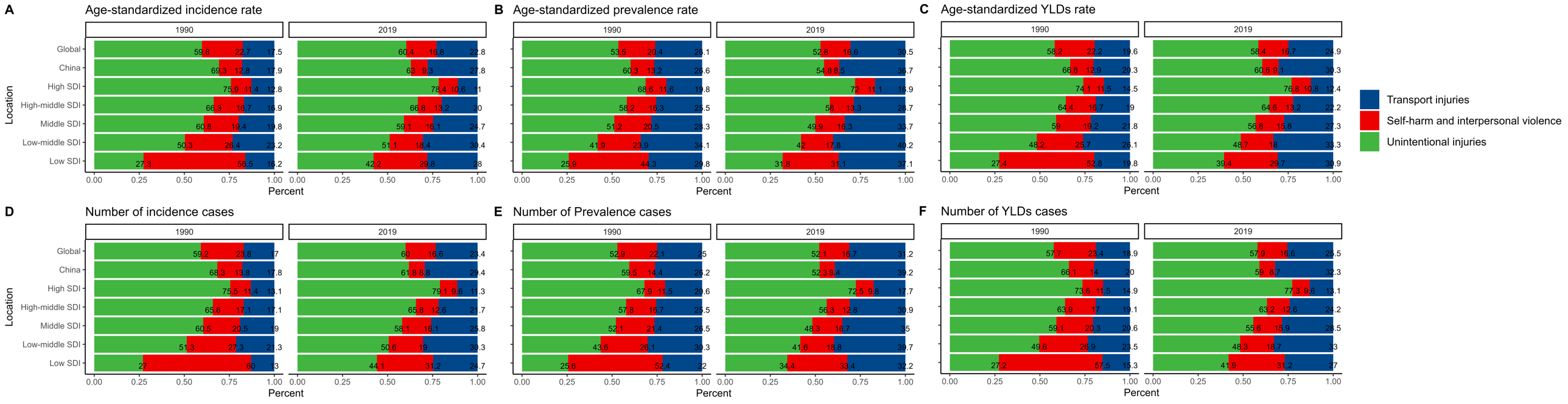

Supplement: Supplementary file 2 — Additional file 2. [file 13049_2024_1258_MOESM2_ESM.zip › additional file 2/Additional file 2 Fig. S7.png]

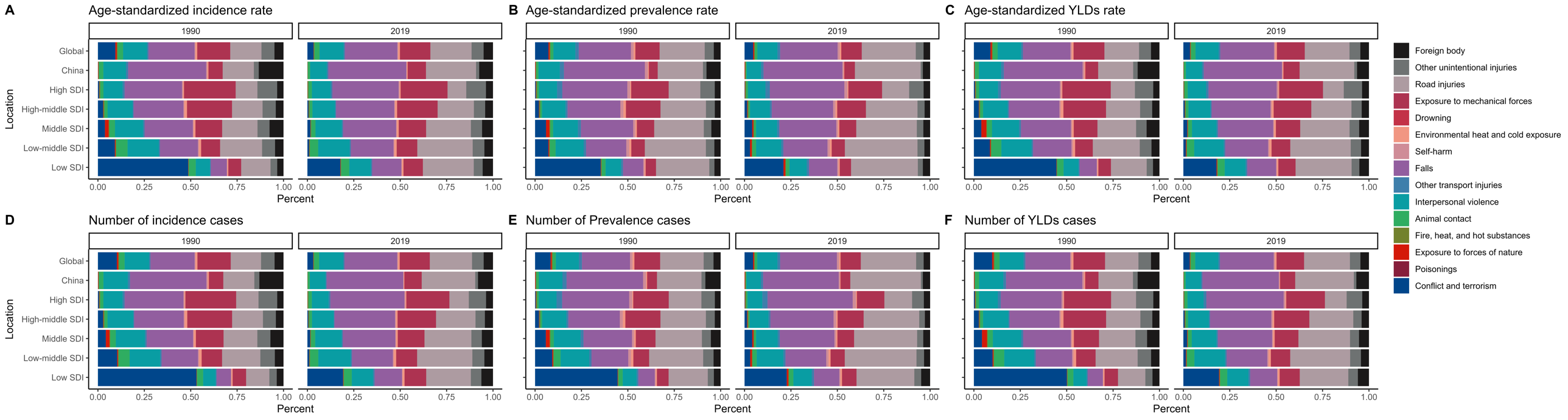

Supplement: Supplementary file 2 — Additional file 2. [file 13049_2024_1258_MOESM2_ESM.zip › additional file 2/Additional file 2 Fig. S8.png]
